# Wearable Walking Assistant for Freezing of Gait With Environmental IoT Monitoring: A Contribution to the Discussion

**DOI:** 10.3389/fpubh.2022.861621

**Published:** 2022-06-20

**Authors:** Rafael A. Bernardes, Filipa Ventura, Hugo Neves, Maria Isabel Fernandes, Pedro Sousa

**Affiliations:** The Health Sciences Research Unit: Nursing (UICISA:E), The Nursing School of Coimbra, Coimbra, Portugal

**Keywords:** Parkinson's disease, freezing of gait, internet of things, self-management, wearables

## Abstract

Parkinson's disease (PD) is the second most common neurodegenerative disease, significantly increasing in the last three decades. Worldwide, seven to ten million people are affected by PD. In people living with PD, freezing of gait (FoG) significantly impacts activities of daily living, potentially leading to falls, injuries, and loss of autonomy. FoG prevalence rates vary widely, reaching at least 50% of patients with PD. Current therapeutic options have limited effectiveness, and their complement with innovative technology-based solutions in the real world is demanded to enhance daily functioning for people living with PD. This article provides a narrative review of current technological developments for people living with PD and, derived from that evidence, presents a perspective on integrating wearable technology and IoT to support telemonitoring and self-management of people living with PD in their daily living environment. Complementing current therapeutic options with technology-based solutions in PD patients' real-world environment is crucial to enhancing the quality of life of people living with PD. In that way, wearable technology and IoT might constitute resources of excellence in seamless monitoring and self-management in people's home environments.

## Introduction

Parkinson's disease (PD) is a neurological disorder with evolving layers of complexity. It has long been characterized by the classical motor features of Parkinsonism associated with Lewy bodies and the loss of dopaminergic neurons in the substantia nigra (1). The parkinsonian symptoms include bradykinesia, muscular rigidity, rest tremor, and postural and gait impairment (2).

In Europe, the estimated prevalence of PD is 1.0% in people with 60 or more years and 3.0% in people older than 80 years, with prevalence rates estimated between 65 and 12.500 per 100.000 and incidence between 5 and 346 per 100.000 person-years (3).

Regarding the global burden of PD, Klietz et al. (3) found a mild increase in caregiver burden in 1 year, highlighting that it is time-consuming and a risk factor for developing depressive symptoms. Economically, PD also has a significant impact worldwide. Yang et al. (4) note a significant economic burden of PD in the United States, with direct medical costs of $25.4 billion and $26.5 billion in indirect and non-medical expenses.

Clinically, a consensus on the classification of PD subtypes has not yet been established. Still, empirical observations suggest two significant subtypes: tremor-dominant PD (with a relative absence of other motor symptoms) and non-tremor-dominant PD (which includes phenotypes described as akinetic-rigid syndrome postural instability gait disorder). People living with PD typically present at least one of four major motor symptoms: Bradykinesia or hypokinesia, resting tremor, rigidity, and postural instability (5). Non-motor symptoms include psychiatric disorders (e.g., hallucinations and delusions, mood disorder), cardiovascular disorders (e.g., orthostatic hypertension, fatigue), neurocognitive disorders, and visual disorders.

**Figure 1:**
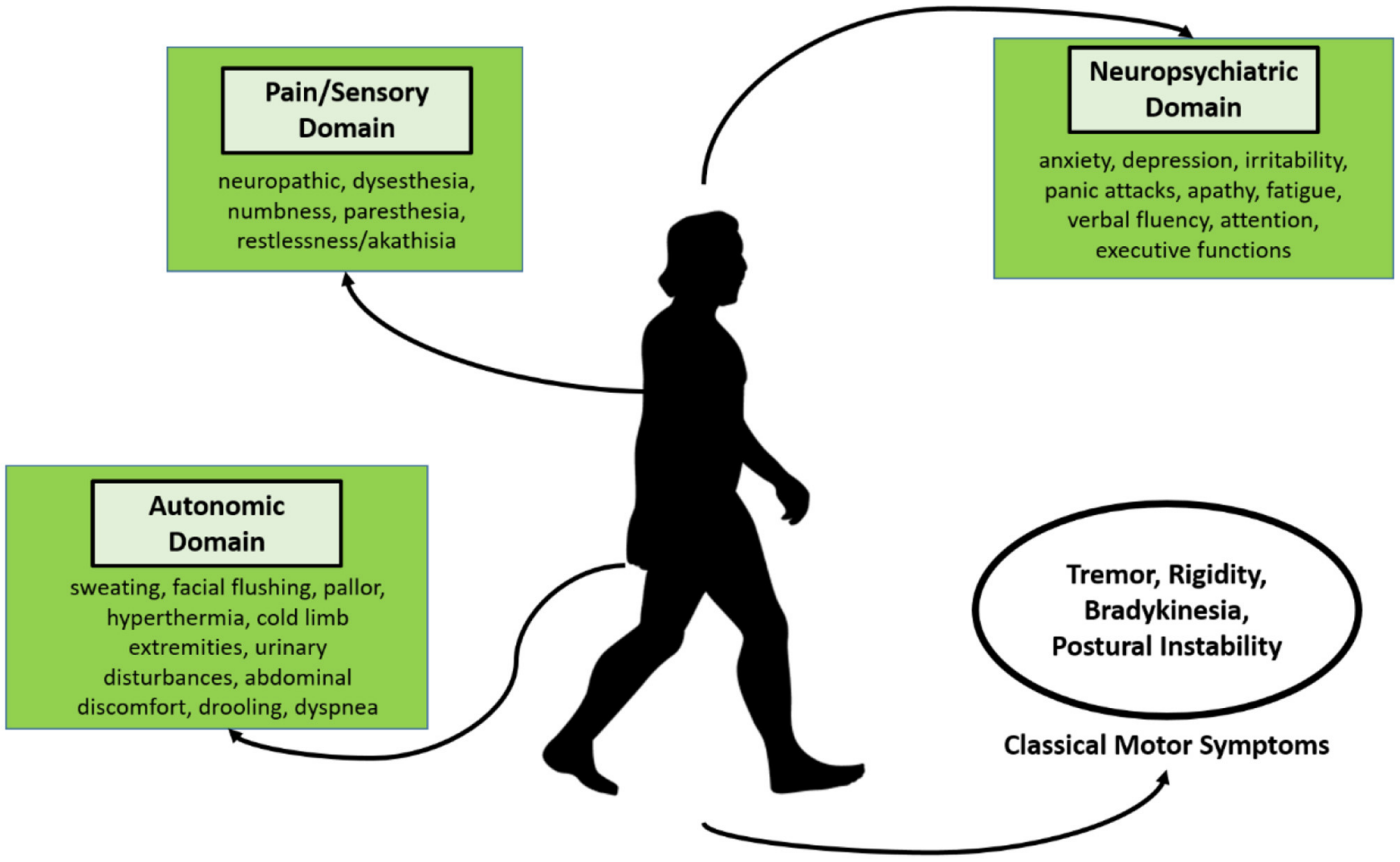
Clinical presentation of PD, according to the motor and non-motor signs and symptoms.

Figure 1 summarizes the main signs and symptoms related to PD. The classic motor symptoms are frequently mentioned by authors (6). PD can also be evidenced by non-motor symptoms, which include psychiatric disorders (e.g., hallucinations and delusions, mood disorder), cardiovascular disorders (e.g., orthostatic hypertension, fatigue), neurocognitive disorders, attention deficit, sexual dysfunction, visual disorders, among others (7, 8). However, a combination of these symptoms poses a more significant impact on the person's quality of life with PD. As for diagnostic purposes, Morgan et al. (9) highlight that diagnostic criteria should also include asymmetry and cogwheel rigidity. Other advances regarding the diagnostic of PD have been observed, with Alpha-synuclein oligomers and small nerve fiber pathology in the skin as potential biomarkers, as well as analysis of presynaptic dopaminergic terminals and the severity of the putamen involvement through ^99m^Tc-TRODAT-1 SPECT Imaging, presenting as potential diagnostic markers of PD (10, 11).

Motor and non-motor signs and symptoms are usually treated using doses of carbidopa/levodopa, depending on the disease's stage. Pharmacological treatment is an important strategy to manage chronic symptoms and increase independence at an initial stage. Yet, several non-pharmacological interventions are available. For example, Church (6) mentioned a technological device, i.e., the red-light-helmet, which uses Light-emitting diodes (LEDs) to alleviate motor symptoms. Morgan et al. (9) state that deep brain stimulation (DBS) is the most effective treatment for motor symptoms. Exercise and neurorehabilitation are increasingly reported as essential measures to fight against motor symptoms. Isernia et al. (12), following the discussion on motor and non-motor interventions, highlight telerehabilitation's efficacy, including physical and cognitive interventions being delivered at a distance.

The social and psychological issues in PD-affected patients should also be considered and might vary in individual patients. Therapies, such as deep brain stimulation and surgical lesioning, should be explored. Further research should be encouraged to better understand the disease's characteristics and etiology. Future scientific research involving Parkinson's disease might enlighten our knowledge of disease onset and progression and deliver some added aspects/components to help find more effective therapies to improve patients' quality of life with PD. As for non-motor symptoms, the latest research trends point to the need to better understand the pathophysiology mechanism before developing new therapeutic approaches (13). However, with pharmacology interventions being insufficient for the management of the person with PD, a clear trend toward developing client-centered interventions that personalize care is becoming more noticeable (14).

According to recent studies, Table 1 provides an overall perspective of the most common pharmacologic and non-pharmacologic treatments for motor and non-motor symptoms (6, 9–15).

**Table 1:** Treatments for motor and non-motor symptoms.

**Symptom domain**	**Associated symptoms**	**Pharmacologic**	**Non-pharmacologic**
Pain/sensory domain	Neuropathic, dysesthesia, numbness, paresthesia, restlessness/akathisia, visual impairments	Anticholinergic, dopamine agonists, trihexyphenidyl, analgesics, opiates	Physical therapy and exercise programs,
Autonomic domain	Sweating, facial flushing, pallor, hyperthermia, cold limb extremities, urinary disturbances, abdominal discomfort, drooling, dyspnea	Fludrocortisone, acetylcholinesterase inhibitors, dopaminergic treatments, anti-muscarinic anticholinergic drugs, β-3 adrenergic agonists	Sex therapy
Neuropsychiatric domain	Anxiety, depression, irritability, panic attacks, apathy, fatigue, verbal fluency, attention, executive functions	Tricyclic antidepressant drugs (TCA), selective serotonin reuptake inhibitors (SSRI), buspirone	Deep brain stimulation (DBS), cognitive behavioral therapy
Others	Sleep disorders, weight loss, malnutrition, osteoporosis, fatigue	Dopamine agonists, gabapentin, pregabalin, vitamin D, calcium	Multiple sleep latency test, mini-nutritional assessment, dual-energy X-ray absorptiometry (DXA), Parkinson's fatigue scale (PFS-16)

As one of the most common and incapacitating PD symptoms that severely compromise patients' quality of life, freezing of gait (FoG) occurs in about 50% of patients with PD (2). Clinically, FoG is characterized by sudden, relatively brief episodes of inability to produce effective forward stepping and typically occur during gait initiation or turning while walking. These gait blocks significantly interfere with daily life, also being on the list of common causes of falls (1). Importantly FoG is now recognized as the leading cause of falling, fracture risk, and activities of daily living disability (16). This risk is compounded because FoG often co-occurs with substantial balance problems and cognitive deficits, mainly frontal executive (17). Associated gait abnormalities affect step calling, step symmetry, and step time consistency before and in-between FoG episodes.

The prevalence of FoG increases with a longer disease duration. It has been reported that 81% of people with PD experienced FoG after a disease duration of 20 years (18). Nieuwboer and Giladi (7) point to four potential mechanisms for FoG events that may explain this time-dependent increase in the prevalence. According to these authors, FoG may occur due to: a motor breakdown associated with the accumulation of various motor deficits (threshold model); an inability to deal with multiple sensory and motor inputs leading to the interruption of locomotion (interference model); behavioral indecision (cognitive model); a cleavage between introductory programming and the intended motor response (decoupling model).

Regardless of the potential model that leads to FoG, the multiple dimensions and systems implicated in this phenomenon highlight FoG management as a significant therapeutic challenge in clinical practice (19). The variety and heterogeneity of existing solutions are a consequence of the complexity of the pathology. However, new and emerging approaches have been developed in recent years, which allow the possibility to enrich and deepen the discussion that has been made in recent times. In this sense, this article intends to contribute to this discussion, highlighting some current gaps and proposing some innovations in the research processes.

## Current Therapeutic Trends and Challenges in FoG Evaluation: The Case of Environmental IoT Monitoring

The most common form of treatment to manage the motor symptoms of PD is Levodopa (LD). LD is the metabolic precursor to dopamine and is used to replace endogenous dopamine at the striatum (2). The medication cycle between two consecutive intakes is roughly divided into two periods, the ON period in which the LD is adequate and the OFF period in which the influence of the medicine has subsided (2). The development of involuntary movements and the ON/OFF phenomenon, i.e., motor response fluctuations uncorrelated with the expectation from the daily medications intake schedule, can limit mobility and complicate dosing.

Gait deficits and FoG are often resistant to pharmacologic treatment. Therefore, effective non-pharmacologic therapies are needed as an adjunct therapy to relieve symptoms and improve mobility (2). There are various approaches to FoG treatment, one of them being non-pharmacological. Previous work has shown that gait performance in PD can be improved by applying continuous external rhythmic auditory, visual or somatosensory cueing (1). Eskofier et al. (19) noted that the constant, automatic monitoring of sensor-based information on walking ability and mobility is increasingly exploited to support objective assessment for preventive and proactive disease management. The Internet of Health Things (IoHT) is remarkably making its way within healthcare interventions and markets worldwide to answer complex and chronic pathological frameworks, just like PD.

As an initial step to further developing IoHT devices, it is vital to understand how IoT works in a healthcare environment, mainly how it contributes to monitoring external environmental stimuli. Usually, IoT consists of a complex connection between devices, machines, and servers with ample data storage (internet), functioning through a network shared between different stakeholders (20). IoT is an exciting topic inserted in the so-called Industrial Revolution 4.0, which aims to connect things and objects anytime and anywhere.

**Figure 2:**
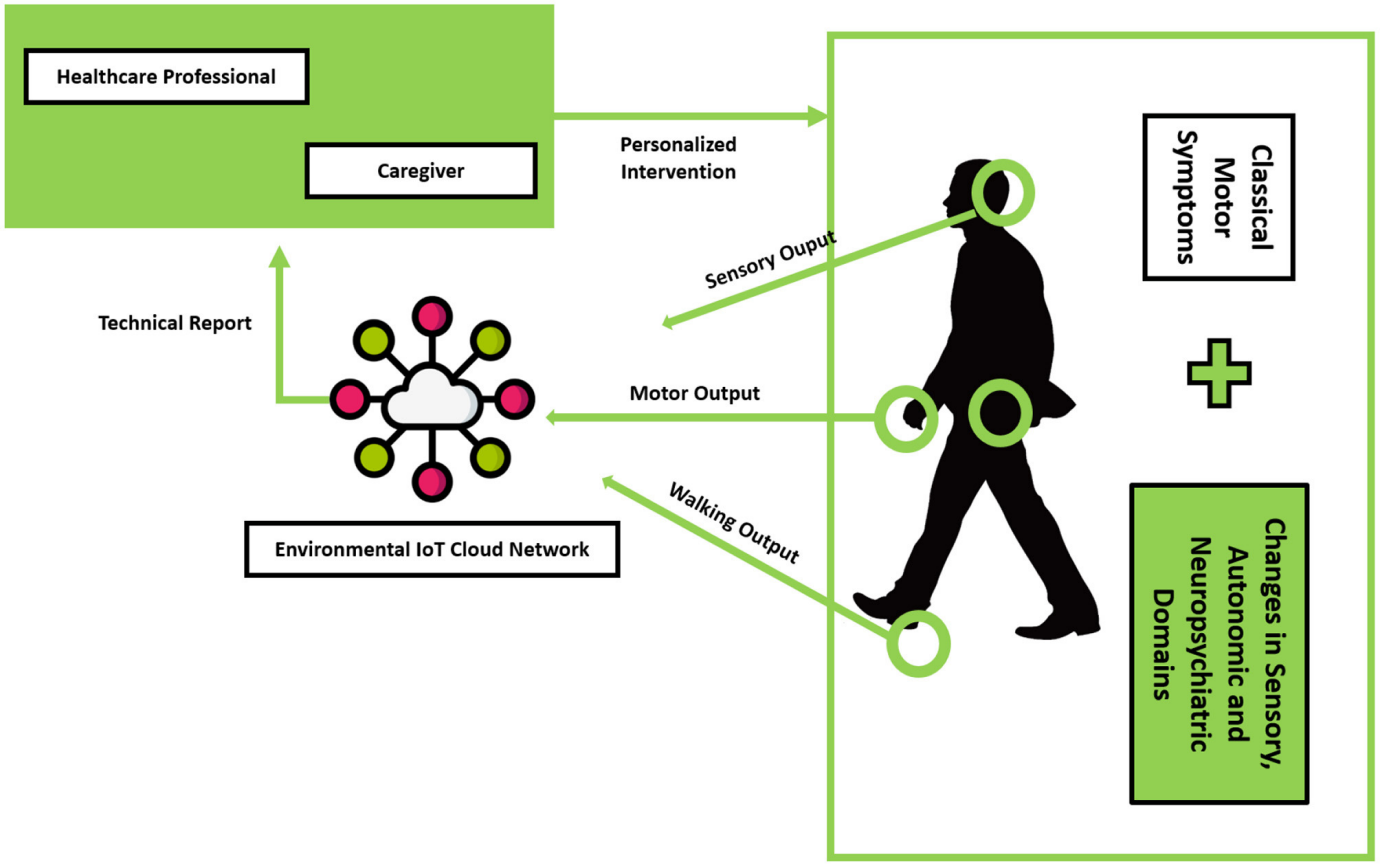
PD management using Environmental IoT Cloud Network.

Following the previous description, we could argue that people with PD are surrounded by several stimuli, internal and external, generating a complex relationship between variables, and making it difficult to perform efficient disease management. This problem is evidenced in Figure 2, with the possible association with Environmental IoT Management.

One of the advantages of Environmental IoT Management is the likely decentralization of the healthcare structure, allowing for a smoother and more efficient interaction between healthcare professionals, caregivers, and patients (21). One of the challenges posed by PD is the chronic and variable nature of signs and symptoms, which are difficult to monitor (21).

## Current Wearable Devices Addressing Freezing of Gait in Parkinson's Disease

A recent systematic review of new assessment methods on PD concludes that there is a particular need for standardized and collaborative studies to confirm the results of preliminary initiatives, assess domains that are currently under-investigated, and better validate the existing and upcoming assessment of PD with technology. Another systematic review (22) has identified and described different wearable insoles with the capacity to recognize patterns, namely stride time, step length, foot clearance, postural sway, gait kinematics, and plantar pressures. Marcante et al. (23) specifically addressed wearable pressure sensors to detect FoG. Some common limitations of these solutions are that their intervention ends in prevention or identification. The ability to associate a personalized intervention by receiving, processing, and sending data with advanced algorithms for health professionals or caregivers is usually not present or requires other devices.

Cueing is a well-established technique that has been shown to improve gait in PD patients in terms of increased walking speed, step length, cadence (total number of steps taken per minute), and reduced number of FoG episodes (24).

Cueing can be defined as using external stimuli which provide temporal (related to time) or spatial (related to space) information to facilitate movement (gait) initiation and continuation. The literature extensively reported three cueing modalities: visual (25), auditory cueing, and somatosensory cueing. The precise mechanism(s) underlying the effectiveness of cueing to ameliorate FoG is unclear. However, previous studies (24) have suggested cueing may compensate for the defective internal rhythm generator of the basal ganglia, consequently affecting the coordination and execution of movement. In this way, PD patients may use auditory, visual, or somatosensory cueing to provide temporal information (i.e., external rhythm) to which movement can be associated. Another hypothesis is that people suffering from PD may use visual cueing to provide spatial information to scale and guide movements, allowing the patient to bypass their defective basal ganglia during gait.

Previous studies (26) have also suggested that cognitive/attentional mechanisms might explain the positive effects of cueing on FoG. Namely, auditory, visual or somatosensory cueing may shift the patient's attention to the task of walking, helping them to consciously think about the next action to be undertaken.

Studies indicate that enhanced proprioceptive information processing could be the mechanism underlying the positive effects of cueing on FoG (27). In this way, the patient may use visual or somatosensory cueing as an artificial means to stimulate the proprioceptive inputs, providing enhanced information on limb position and movement during gait.

Table 2 summarizes current devices and registered patents on cueing technology to treat FoG, describing the main trends on this topic.

**Table 2:** Current cueing devices and registered patents for FoG treatment.

**Authors**	**Device**	**Method and description**	**Performance**
Mazilu et al. (28)	GaitAssist	Personalized wearable system for FoG support and gait training in unsupervised environments. The two functionalities of the GaitAssist are (1) gait support during daily-life activities to avoid or reduce FoG episodes; (2) training support—as a personal assistant for the gait exercises. It provides audio feedback at critical moments throughout walking when FoG appears.	• Tested with five patients; • FoG real-time hit rate was equal to 97% (99 out of 102), with a detection delay of 0.25s; • FoG events shorter than that period could not be detected; • A decision in the algorithm regarding the FoG event is performed in at most 1 ms. • The system can be continuously used in the assistive mode for up to around 4 h.
Bächlin et al. (2)	Wearable Assistant for Online FoG Detection	A tiny computer for recording data and online signal processing. Customized platform based on an Intel XScale family processor and uses a Linux operating system.	• The device successfully identified 237 FoG events in eight patients (0–66 per patient). • The length of the FoG events ranged from 0.5 to 40.5 s, and over 50% of the FoG episodes lasted longer than 5 s. • Specificity ranged from 39.7 to 88.9%, and sensitivity from 34.1 to 99%.
Van Gerpen (29)	System and method for alleviating freezing gait and gait hypokinesia in users with extrapyramidal disorders	Visual cue for a user of a walker or walking aid device. Provide a constant or recurring stimulus to reduce or substantially eliminate the occurrence of FoG, gait hypokinesia, or stride reduction in a user, such as one suffering from parkinsonism.	Patent (n/a)
Buated (5) McCandless (30)	LaserCane	External cues improve walking ability in PD patients, stating that it significantly enhances FoG, specifically by increasing patients' stride length and velocity immediate improvements during gait initiation when using the Laser Cane over other interventions.	• Buated et al. (5) applied an external cue with a laser cane in 30 patients, significantly reducing time, the number of freezes (steps) from 0.33 ± 0.84 to 0, and increasing stride length (cm) from 6.82 ± 18.54 to 90.05 ± 19.44. • McCandless et al. (28), in their study with 20 participants, the visual cue with the laser cane reduced the percentage of freezing episodes from 81.58 to 27.50%, increasing velocity (m/s) from 0.335 to 0.455.
Mcloul et al. (31)	Movement initiation device used in Parkinson's disease and other disorders which affect muscle control	A portable wearable device produces rhythmic stimuli in auditory, visual, tactile, and/or vibratory activity that initiates or assists in the continuing movement of the body's muscles that tend to become rigid and immobile. Includes a housing, a clinician-accessible controller, a user-accessible controller, a transducer for producing sound or vibratory signals, and a computer interface.	Patent (n/a)
Shim et al. (32)	Walking assistance method and apparatus	Walking assistance method and apparatus, in detail, a control device that may estimate a gait motion of a user based on pressure data indicating information on a pressure applied to a sole of the user, and provide a feedback corresponding to the gait motion to the user by controlling a vibrator to apply vibration to the sole of the user, is provided.	Patent (n/a)
Akay (33)	Intelligent wearable monitor systems and methods	An intelligent wearable monitoring system includes a wireless personal area network to monitor a patient's motor functions. The private wireless network consists of a smart accelerometer unit, a personal server, and a remote access unit. The intelligent accelerometer unit measures the acceleration data of the patient in real-time. Motor function information is transmitted to a remote access unit for statistical analysis and formatting into visual representations.	Patent (n/a)
Chun et al. (34)	Wearable vibratory stimulation device and operational protocol thereof	The wearable stimulation device includes a measuring instrument for obtaining data relating to a body motion of a user who wears the vibratory stimulation device, a walking pattern database for storing normal walking pattern data collected by measuring general persons having standard walking patterns, and information about an inherent walking pattern analysis result of a specific user, a controller for analyzing the body motion information of the user, transmitted from the measuring instrument.	Patent (n/a)
Miyake (35)	Walking Aid System	The walking aid system comprised of a sensor section for sensing the motion rhythm of a walker, a recording section for recording values of measurements of the motion rhythm felt with the sensor section, a target setting section for setting a target value for the motion rhythm of the walker, a timing generating section for generating a timing signal according to the difference between the measurement and the target value, and a stimulus generating section for generating rhythm stimulus that is recognizable by the walker, according to the timing signal generated with the timing generating section.	Patent (n/a)

Although several wearable devices have increased for patients with Parkinson's, few seem to use technologies such as environmental IoT, as described and defined earlier in this article. It is interesting that some projects and devices mentioned in Table 2 use techniques such as health education and learning, namely by training specific exercises. Others have developed stimulation devices with a concrete therapeutic objective. This type of resource—education for health, day-to-day devices—due to its proximity and usability for the end-user, is a competitive advantage among the various devices developed or under development.

Despite this, and given the novelty of environmental IoT, particularly the collection of data, learning of personal patterns, machine learning, and generation of outputs personalized to the context, some projects and devices manage to fulfill the proposed purpose. Still, they would have the capacity to be more effective and satisfactory for people if they integrated all systems discussed so far.

Thus, the reflection proposed here contributes to broadening the discussion on the subject and revealing the importance of making fundamental and clinical investigations interdisciplinary, proposing more cooperation and synergies between the various disciplines of knowledge. In the case of PD, the participation of nurses in the construction of medical projects and devices can be very significant due to their thorough understanding of the person's adaptive capacity to the health-disease situation being experienced. But, it is not only nursing which benefits from cooperation with robotics, electronics, mechanics, medicine, and occupational therapy, among others.

## Intelligent Shoes for Digital Health Management

Generally, wearable health assistants aim to reduce the number and length of motor blocks, thus increasing safety while walking (2). Even assuming that this is a core function of the developed devices, Maetzler (36) indicates that, currently, there are no technology-based tools available that: (a) provide valid and accurate parameters of clinically relevant features of PD; (b) provide evidence of an ecologically relevant effect on specific clinical applications; (c) a definition of a target range, wherein the parameter reflects the adequate treatment response; (d) simple implementation allowing for repetitive use.

Within this scope, the development of smart shoes for managing complex health conditions is not new (37). Namely, this technology has already been applied to situations like degenerative spinal cord disabilities (37), frailty syndrome (38), diabetes (39), or even glaucoma (40). In terms of non-pharmacological treatment, previous work has shown that gait performance in PD can be improved by applying continuous external rhythmic auditory, visual or somatosensory cues (1). Accordingly, various behavioral “tricks” were developed by clinicians and patients to overcome freezing events. These tricks include marching to command, stepping over a walking stick or cracks in the floor, walking to music or a beat, and shifting body weight.

Mobility monitoring technologies demand research in sensor-based data acquisition and subsequent analysis to support objective and clinically relevant gait analysis (41). To undergo research in this field, Eskofier et al. (19) have already bulleted the main elements to take into consideration:

1) Remote gait assessment with smart shoes requires a data acquisition system that will collect sensor data;
2) An efficient management of power is essential to reduce the frequency of required charging of devices.

The variety of sensors that are applicable in the context of smart shoes are:

- Relative location and orientation determination using inertial-magnetic measurement units (e.g., accelerometer, gyroscope, magnetometer);
- Absolute location determination using satellite navigation systems (e.g., GPS, Glonass, Galileo);
- Foot plantar pressure determination using various forms of pressure sensors, which provide information regarding how effectively and efficiently individuals control the distribution of the body weight during gait;
- Ambient environmental sensors, such as atmospheric pressure sensors for altitude-dependent activities (e.g., stair climbing), light and sound sensors;
- Two major applications of algorithmic methods are to be used:
- Activity pattern recognition;
- Gait signature derivation for medical diagnostic and treatment contexts.

When we mention “smart shoes,” we are necessarily restricting the discussion to intelligent footwear, but, in a broader sense, to wearable devices which support people with PD during walking. Such technology is not new nor under-studied, mainly because of the importance of FoG treatment, as stated before. The application of wearable walking assistants can include several features and present different treatment goals. For example, Pardoel et al. (42) have employed a conjoint precision analysis of PD motor symptoms using IMU and plantar pressure, successfully identifying Total-FoG (pre-FoG, FoG Transition, and FoG). The authors conclude that the developed system could lead to appropriate prevention of freeze or help to exit the episode. This is an example of an early detection and prevention wearable assistant device.

For PD, it is utterly important, at some point, to estimate gait patterns and recognize important gait events, particularly FoG subtypes. In this sense, Eskofier et al. (19) state that gait event recognition technologies compare the incoming sensor data, preprocessed time-series or computed gait features to reference characteristics of important gait events.

## European Projects: An Effort Toward an Effective Solution

In the last years, there has been an effort to develop and structure new interventions and technological devices to manage and treat FoG, which can be found in the European database CORDIS (cordis.europa.eu).

Main technological advances have been focused on wireless technologies with high-resolution tracking of gait patterns in PD patients, dimensioning time-varying biomechanics related to locomotion, like the project “*Decoding impairments of gait and balance from local field potentials in patients with Parkinson's disease (gaitCODE)*.”

Some projects were interested in developing portable devices for people living with PD, delivering non-invasive neurostimulation signals, as in the *Automated Mechanical Peripheral Stimulation for motor rehabilitation in people living with Parkinson's Disease (GONDOLA)*, which specifically provided physical neurostimulations on specific points in both feet. These neurological stimuli allow for increasing afferent inputs from the peripheral nervous system to the spinal cord, inducing a better functioning of the central pattern generator (the mechanism that regulates movements in the body). Similarly, *Industrial Academic Initial Training Network toward the focused treatment of age-related motor symptoms* project aims to study specific basal ganglia pathways and stimulate balance and postural control through virtual reality, body-worn movement monitors, therapeutic cues, and individualized training.

Portable and wearable devices seem to be the most common strategy to address FoG, particularly when it is necessary to monitor related motor symptoms in real-time, to support clinical decisions. This is the case of the project *Unobtrusive, Continuous and Quantitative Assessment of Parkinson's disease: Hard Evidence for Optimal Disease Management with Information Technologies (Stat-On TM, Park-IT)*, which developed a small wearable device to continuously monitor movement patterns, being total autonomous and comfortably worn in patient's waist. An exciting and noteworthy addition is the possibility to transfer the motor assessment data to an external mobile device, which then, through machine learning, provides an identification of specific PD symptoms.

Treatment-related projects have also been developed, like a *Closed-loop system for personalized and at-home rehabilitation of people with Parkinson's Disease (CuPiD)*, designed to meet optimal rehabilitation of PD patients with personalized training at home. It's based on an ICT-enabled solution, with a tailored solution to target mobility, cognitive function, and debilitating symptoms like FoG.

The way has been drawn to reach a more person-oriented treatment, with the ultimate goal of achieving autonomy and independence. This is the project's logic *PROPHETIC: An innovative personal Healthcare Service for holistic remote management and treatment of Parkinson's patients*, which exploits miniature information systems to manage the disease, with remote and continuous monitorization of patients and sharing management plan data between caregivers and health professionals. This project, similarly to others, applied virtual reality to enhance treatment with gamification features at home.

Other projects, like the one developed by *Kinetikos* or *TecaPark*, in addition to presenting the features of the previous projects, include a more subjective assessment with a biopsychosocial dimension and address quality of life as part of the treatment of FoG.

## Discussing Future Trends

With IoT and the increase in the ability to computationally collect and process data, devices anchored in this technology will quickly become part of our daily living activities. Thus, it is no surprise that most client-centered devices developed a focus on wearable technology. However, this aspect adds a layer of complexity, as questions of comfort, usability, and aesthetics need to be considered.

With this shift to a self-care paradigm, where the relationship between client and healthcare provider will change, the key to developing future viable solutions will imply including dimensions that were not previously considered when using technology. The presence of wearable devices in patients experiencing FoG will most certainly become a reality, with the client as an active part in demanding this technology and choosing which option fits their personal preferences (43). Among the wearable devices that will become mandatory for people with PD experiencing FoG, smart shoes will most certainly be one, if not the most present technology (44).

The potential associated with smart shoes is immense. Not only will PD patients be able to understand what happens before and during the FoG event, but the data collected throughout the whole process will help researchers and healthcare providers develop personalized interventions and new technologies, like domotics, that will significantly decrease the burden associated with FoG (45).

Smart shoes will most certainly be data-collection-based. The potential to help the patient to focus the attention on the task of walking by restoring internal cueing and internal driving is a dimension that can be associated with a machine-learning algorithm with the potential to significantly reduce the risks associated with the FoG (46).

These devices will further assist healthcare providers and researchers in understanding how the real-world environment influences controlled interventions. Until now, clinical research has been associated with limited control of variables. Data collected during clinical trials represent only part of how people deal with their health conditions and how the intervention affects (positively or negatively) the client's quality of life.

Data collection will become more complex, with IoHT allowing machine-learning algorithms to help the client understand what is happening in real-time (47, 48). They will also help address FoG causes and consequences by preventing them through the individualized establishment of cueing strategies and alert systems that will allow a quicker and better response to a fall, namely through an intelligent environment (49).

Future trends portray the need to engage all the actors in this phenomenon: the FoG. Understanding how wearable technology can be part of this new self-care paradigm will significantly impact how patients deal with this condition. Thus, assuring citizen engagement in developing IoHT strategies will be a requirement for every device, with new variables being integrated into how these are built (50).

## Conclusions

FoG is a complex episodic motor symptom that significantly affects the quality of life and activities of daily living. Despite the existing pharmacological treatments to alleviate PD symptoms, namely tremors and bradykinesia, there are limited resources to treat FoG. Accordingly, technology has been consecutively applied, concomitant with conventional approaches, namely external cueing, either visual, auditory, or somatosensory.

This perspective successfully presented and described current therapeutic options in PD, eliciting their advantages and limitations. One of the major conclusions is that complementing the standard treatment with innovative technology-based solutions is helpful for enhancing the daily life of people with PD. Wearable technology and IoT might constitute important assets for monitoring and self-management in homecare. Existent solutions still need further development to address the need to deliver a personalized intervention by receiving, processing, and sending data with advanced algorithms for health professionals or caregivers.

Regarding cueing devices, one of the most recent and modern approaches is wearable technology embedded in footwear, which increases neurological stimuli in PD patients and thereby their motor functions. Smart shoes have been developed for many pathologic signs and symptoms and are currently being tested for PD.

State of the art aims to apply IoHT and virtual reality to monitor patients and increase their autonomy, also propelling caregiver abilities through personalized programs. Future trends seem to be reaching a person-centered approach, focusing on comfort, usability, and aesthetics, namely in footwear development.

Some limitations have been identified: the unsystematic review might have implied the loss of previous important works on the topic; the focus on wearable shoes provided a more narrowed review, thus contributing to less enriched work.

## Data Availability Statement

The original contributions presented in the study are included in the article/supplementary material, further inquiries can be directed to the corresponding author.

## Author Contributions

RB and PS led the design of the manuscript. HN and FV assisted in drafting the manuscript. MF provided advice on key study issues. All authors contributed equally to the narrative literature review and evidence synthesis. All authors contributed important intellectual content to the manuscript and approved the final version for publication.

## Funding

This work was funded by National Funds through the FCT—Foundation for Science and Technology, I.P., within the scope of the project Refª. UIDB/00742/2020. The work of FV was funded by the Portuguese Foundation for Science and Technology (FCT), CEECINST/00103/2018. The funder had no role in the study.

## Conflict of Interest

The authors declare that the research was conducted in the absence of any commercial or financial relationships that could be construed as a potential conflict of interest.

## Publisher's Note

All claims expressed in this article are solely those of the authors and do not necessarily represent those of their affiliated organizations, or those of the publisher, the editors and the reviewers. Any product that may be evaluated in this article, or claim that may be made by its manufacturer, is not guaranteed or endorsed by the publisher.

## References

[1] Bächlin M Plotnik M Roggen D Maidan I Hausdorff JM Giladi N. Wearable assistant for Parkinson's disease patients with the freezing of gait symptom. IEEE Trans Inf Technol Biomed. (2010) 14:436–46. 10.1109/TITB.2009.2036165 19906597

[2] Bächlin M Plotnik M Roggen D Giladi N Hausdorff JM Tröster G. Wearable system to assist walking of Parkinson's disease patients. Methods Inf Med. (2010) 49:88–95. 10.3414/ME09-02-0003 20011807

[3] Klietz M von Eichel H Schnur T Staege S Höglinger GU Wegner F. One year trajectory of caregiver burden in Parkinson's disease and analysis of gender-specific aspects. Brain Sci. (2021) 11:295. 10.3390/brainsci11030295 33652825 PMC7996933

[4] Yang W Hamilton JL Kopil C Beck JC Tanner CM Albin RL. Current and projected future economic burden of Parkinson's disease in the US. NPJ Parkinsons Dis. (2020) 6:15. 10.1038/s41531-020-0117-1 32665974 PMC7347582

[5] Buated W Sriyudthsak M Sribunruangrit N Bhidayasiri R. A low-cost intervention for improving gait in Parkinson's disease patients: a cane providing visual cues. Eur Geriatr Med. (2012) 3:126–30. 10.1016/j.eurger.2012.01.006

[6] Church FC. Treatment options for motor and non-motor symptoms of Parkinson's disease. Biomolecules. (2021) 11:612. 10.3390/biom11040612 33924103 PMC8074325

[7] Kuresan H Samiappan D Ghosh S Gupta AS. Early diagnosis of Parkinson's disease based on non-motor symptoms: a descriptive and factor analysis. J Ambient Intell Humaniz Comput. (2021). 10.1007/s12652-021-02944-0

[8] Berganzo K Tijero B González-Eizaguirre A Somme J Lezcano E Gabilondo I. Motor and non-motor symptoms of Parkinson's disease and their impact on quality of life on different clinical subgroups. Neurol. (2016) 31:589–91. 10.1016/j.nrleng.2014.10.016 25529173

[9] Morgan JC Fox SH. Treating the motor symptoms of Parkinson disease. Continuum. (2016) 22:1064–85. 10.1212/CON.0000000000000355 27495198

[10] Vacchi E Senese C Chiaro G DIsanto G Pinton S Morandi S. Alpha-synuclein oligomers and small nerve fiber pathology in skin are potential biomarkers of Parkinson's disease. NPJ Parkinsons Dis. (2021) 7:119. 10.1038/s41531-021-00262-y 34930911 PMC8688481

[11] Sasannezhad P Juibary AG Sadri K Sadeghi R Sabour M Kakhki VRD. ^99m^Tc-TRODAT-1 SPECT imaging in early and late onset Parkinson's disease. Asia Ocean J Nucl Med Biol. (2017) 5:114–9. 10.22038/aojnmb.2017.8844 28660222 PMC5482916

[12] Isernia S Tella SD Pagliari C Jonsdottir J Castiglioni C Gindri P. Effects of an innovative telerehabilitation intervention for people with Parkinson's disease on quality of life, motor and non-motor abilities. Front Neurol. (2020) 11:846. 10.3389/fneur.2020.00846 32903506 PMC7438538

[13] Tibar H Bayad KE Bouhouche A Haddou EHAB Benomar A Yahyaoui M. Non-motor symptoms of Parkinson's disease and their impact on quality of life in a cohort of Moroccan patients. Front Neurol. (2018) 9:170. 10.3389/fneur.2018.00170 29670566 PMC5893866

[14] Jagadeesan AJ Murugesan R Devi SV Meera M Madhumala G Padmaja MV. Current trends in etiology, prognosis and therapeutic aspects of Parkinson's disease: a review. Acta Biomed. (2017) 88:249–62. 10.23750/abm.v88i3.6063 29083328 PMC6142835

[15] Gupta S Shukla S. Non-motor symptoms in Parkinson's disease: opening new avenues in treatment. Curr Opin Behav Sci. (2021) 2:100049. 10.1016/j.crbeha.2021.100049

[16] Nonnekes J Snijders AH Nutt JG Deuschl G Giladi N Bloem BR. Freezing of gait: a practical approach to management. Lancet Neurol. (2015) 14:768–78. 10.1016/S1474-4422(15)00041-1 26018593

[17] Gan J Liu W Cao X Xie A Li W Yuan C. Prevalence and clinical features of FOG in Chinese PD patients, a multicenter and cross-sectional clinical study. Front Neurol. (2021) 12:568841. 10.3389/fneur.2021.568841 33763009 PMC7982534

[18] Nieuwboer A Giladi N. Characterizing freezing of gait in Parkinson's disease: models of an episodic phenomenon. Mov Disord. (2013) 28:1509–19. 10.1002/mds.25683 24132839

[19] Eskofier BM Lee SI Baron M Simon A Martindale CF Gabner H. An overview of smart shoes in the internet of health things: gait and mobility assessment in health promotion and disease monitoring. App Sci. (2017) 7:986. 10.3390/app7100986

[20] Sunny AI Zhao A Li L Sakiliba SK. Low-cost IoT-based sensor system: a case study on harsh environmental monitoring. Sensors. (2021) 21:214. 10.3390/s21010214 33396278 PMC7795175

[21] Pasluosta CF Gassner H Winkler J Klucken J Eskofier BM. An emerging era in the management of Parkinson's disease: wearable technologies and the internet of things. IEEE J Biomed Health Inform. (2015) 19:1873–81. 10.1109/JBHI.2015.2461555 26241979

[22] Channa A Popescu N Ciobanu V. Wearable solutions for patients with Parkinson's disease and neurocognitive disorders: a systematic review. Sensors. (2020) 20:2713. 10.3390/s20092713 32397516 PMC7249148

[23] Marcante A Marco RD Gentile G Pellicano C Assogna F Pontieri 0046E. Foot pressure wearable sensors for freezing of gait detection in Parkinson's disease. Sensors. (2021) 21:128. 10.3390/s21010128 33379174 PMC7794778

[24] Caudron S Guerraz M Eusebio A Gros JP Azulay JP Vaugoyeau M. Evaluation of a visual biofeedback on the postural control in Parkinson's disease. Neurophysiol Clin. (2014) 44:77–86. 10.1016/j.neucli.2013.10.134 24502908

[25] Young WR Shreve L Quinn EJ Craig C Bronte-Stewart H. Auditory cueing in Parkinson's patients with freezing of gait. What matters most: action-relevance or cue-continuity? Neuropsychologia. (2016) 87:54–62. 10.1016/j.neuropsychologia.2016.04.034 27163397

[26] Bunting-Perry L Spindler M Robinson KM Noorigian J Cianci HJ Duda JE. Laser light visual cueing for freezing of gait in Parkinson disease: a pilot study with male participants. J Rehabil Res Dev. (2013) 50:223–30. 10.1682/JRRD.2011.12.0255 23761003

[27] Bekkers EM Dockx K Heremans E. The contribution of proprioceptive information to postural control in elderly and patients with Parkinson's disease with a history of falls. Front Hum Neurosci. (2014) 8:939. 10.3389/fnhum.2014.00939 25505395 PMC4241823

[28] Gerpen V. System and Method for Alleviating Freezing Gait and Gait Hypokinesia in Users with Extrapyramidal Disorders (2015).

[29] McCandless PJ Evans BJ Janssen J Selfe J Churchill A Richards J. Effect of three cueing devices for people with Parkinson's disease with gait initiation difficulties. Gait Posture. (2016) 44:7–11. 10.1016/j.gaitpost.2015.11.006 27004625 PMC4863931

[30] Mcloul RF Oberman C. Movement Initation Device Used in Parkinson's Disease and Other Disorders Which Affect Muscle Control (2002).

[31] Shim Y Lee J Park Y Ahn S Hyung S. Walking Assistance Method and Apparatus. World International Property Organization (WIPO).

[32] Akay M. Intelligent Wearable Monitor Systems and Methods. Munich: European Patent Office (EPO). (2005).

[33] Chun CM Kim CH Choi JH Kim SJ. Wearable Vibratory Stimulation Device and Operational Protocol Thereof. World International Property Organization (WIPO). (2014).

[34] Miyake Y. Walking Aid System. World International Property Organization (WIPO). (2011).

[35] Maetzler W Klucken J Horne M. A clinical view on the development of technology-based tools in managing Parkinson's disease. Mov Disord. (2016) 31:1263–71. 10.1002/mds.26673 27273651

[36] Lee SI Park E Huang A Mortazavi B Garst JH Jahanforouz N. Objectively quantifying walking ability in degenerative spinal disorder patients using sensor equipped smart shoes. Med Eng Phys. (2016) 38:442–9. 10.1016/j.medengphy.2016.02.004 26970892 PMC6470359

[37] Schwenk M Mohler J Wendel C D'Huyvetter K Fain M Taylor-Piliae R. Wearable sensor-based in-home assessment of gait, balance, and physical activity for discrimination of frailty status: baseline results of the Arizona frailty cohort study. Gerontology. (2015) 61:258–67. 10.1159/000369095 25547185 PMC4452118

[38] Perrier A Vuillerme N Luboz V Bucki M Cannard F Diot B. Smart diabetic socks: embedded device for diabetic foot prevention. IRBM. (2014) 35:72–6. 10.1016/j.irbm.2014.02.004

[39] Ma Y Amini N Chasemzadeh H. Wearable sensors for gait pattern examination in glaucoma patients. Microprocess Microsyst. (2016) 46:67–74. 10.1016/j.micpro.2016.07.001

[40] Benz HL Caldwell B Ruiz JP Saha A Ho M Christopher S. Patient-centered Parkinson's disease. MDM Policy Pract. (2021) 6:23814683211021380. 10.1177/23814683211021380 34277950 PMC8255597

[41] Sánchez-Ferro Á Elshehabi M Godinho C Salkovic D Hobert MA Domingos J. New methods for the assessment of Parkinson's disease (2005 to 2015): a systematic review. Mov Disord. (2016) 31:1283–92. 10.1002/mds.26723 27430969

[42] Pardoel S Shalin G Nantel J Lemaire ED Kofman J. Early detection of freezing of gait during walking using inertial measurement unit and plantar pressure distribution DATA. Sensors. (2021) 21:2246. 10.3390/s21062246 33806984 PMC8004667

[43] Reina-Bueno M Calvo-Lobo C López-López D Palomo-López P Becerro-de-Bengoa-Vallejo R. Effect of foot orthoses and shoes in Parkinson's disease patients: a PRISMA systematic review. J Pers Med. (2021) 11:1136. 10.3390/jpm11111136 34834488 PMC8621527

[44] Simonet C Noyce AJ. Domotics, smart homes, and Parkinson's disease. J Parkinsons Dis. (2021) 11:S55–63. 10.3233/JPD-202398 33612494 PMC8385512

[45] Muthukrishnan N Abbas JJ Shill HA Krishnamurthi N. Cueing paradigms to improve gait and posture in Parkinson's disease: a narrative review. Sensors. (2019) 19:5468. 10.3390/s19245468 31835870 PMC6960538

[46] Gómez J Oviedo B Zhuma E. Patient monitoring system based on internet of things. Proc Comp Sci. (2016) 83:90–7. 10.1016/j.procs.2016.04.103

[47] Chiuchisan I Costin H-N Geman O. Adopting the internet of things technologies in health care systems. IEEE. (2014) 532–5. 10.1109/ICEPE.2014.6969965

[48] Uddin MS Alam JB Banu S. Real time patient monitoring system based on internet of things. IEEE. (2017) 2017:516–21. 10.1109/ICAEE.2017.8255410

[49] Sweeney D Quinlan LR Browne P Richardson M Meskell P ÓLaighin G. Technological review of wearable cueing devices addressing freezing of gait in Parkinson's disease. Sensors. (2019) 19:1277. 10.3390/s19061277 30871253 PMC6470562

[50] Mazilu S Blanke U Hardegger M Tröster G Gazit E Dorfman M. GaitAssist: a wearable assistant for gait training and rehabilitation in Parkinson's disease. ACM Trans Interact Intell Syst. (2015) 5:1–31. 10.1145/2701431

